# Crystal structure and biophysical characterization of the nucleoside diphosphate kinase from *Leishmania braziliensis*

**DOI:** 10.1186/s12900-015-0030-8

**Published:** 2015-02-03

**Authors:** Plínio Salmazo Vieira, Priscila Oliveira de Giuseppe, Mario Tyago Murakami, Arthur Henrique Cavalcante de Oliveira

**Affiliations:** Laboratório Nacional de Biociências (LNBio), Centro Nacional de Pesquisa em Energia e Materiais (CNPEM), Campinas, SP Brazil; Departamento de Química, Faculdade de Filosofia Ciências e Letras de Ribeirão Preto, Universidade de São Paulo, Ribeirão Preto, SP Brazil; Rua Giuseppe Máximo Scolfaro, 10000, Pólo II de Alta Tecnologia de Campinas, Post office box 6192, Zip code: 13083-970 Campinas, SP Brazil; Avenida Bandeirantes, 3900, Monte Alegre, Zip Code 14040-901 Ribeirão Preto, SP Brazil

**Keywords:** Nucleoside diphosphate kinase, *Leishmania braziliensis*, Quaternary structure, Conformational stability

## Abstract

**Background:**

Nucleoside diphosphate kinase (NDK) is a housekeeping enzyme that plays key roles in nucleotide recycling and homeostasis in trypanosomatids. It is also secreted by the intracellular parasite *Leishmania* to modulate the host response. These functions make NDK an attractive target for drug design and for studies aiming at a better understanding of the mechanisms mediating host-pathogen interactions.

**Results:**

We report the crystal structure and biophysical characterization of the NDK from *Leishmania braziliensis* (*Lb*NDK). The subunit consists of six α-helices along with a core of four β-strands arranged in a β2β3β1β4 antiparallel topology order. In contrast to the NDK from *L. major,* the *Lb*NDK C-terminal extension is partially unfolded. SAXS data showed that *Lb*NDK forms hexamers in solution in the pH range from 7.0 to 4.0, a hydrodynamic behavior conserved in most eukaryotic NDKs. However, DSF assays show that acidification and alkalization decrease the hexamer stability.

**Conclusions:**

Our results support that *Lb*NDK remains hexameric in pH conditions akin to that faced by this enzyme when secreted by *Leishmania* amastigotes in the parasitophorous vacuoles (pH 4.7 to 5.3). The unusual unfolded conformation of *Lb*NDK C-terminus decreases the surface buried in the trimer interface exposing new regions that might be explored for the development of compounds designed to disturb enzyme oligomerization, which may impair the important nucleotide salvage pathway in these parasites.

## Background

Leishmaniases are classified according to their clinical manifestations as cutaneous, mucocutaneous, visceral and post kala-azar dermal [1]. These diseases are endemic in 98 countries on five different continents, threatening about 350 million people and being considered a public health problem [2]. They are caused by flagellate protozoa from the genus *Leishmania*, which are transmitted to humans and other mammals by sandflies. In the mammalian host, *Leishmania* spp. infect macrophages, thus being studied not only as the causative agents of leishmaniases, but also as a model for intracellular parasitism [3].

Promising targets for drug design and discovery against leishmaniases include enzymes involved in fundamental metabolic pathways for these parasites such as nucleoside diphosphate kinases (NDKs) (EC 2.7.4.6) [4]. NDKs catalyze the transfer of the γ-phosphoryl group from a nucleoside triphosphate donor to a nucleoside diphosphate acceptor [5], using a ping-pong mechanism involving a phosphohistidine intermediate [6]. The protein is considered a housekeeping enzyme and is essential for the maintenance of intracellular NTP levels [7,8]. Eukaryotic NDKs have been associated with several biological processes such as G proteins regulation [9-11], polysaccharide synthesis [12], cell elongation [13] and gene transcription [14].

In pathogenic microorganisms, additional roles are proposed for secreted NDKs, including modulation of host purinergic signaling and attenuation of reactive oxygen species production [15]. *Leishmania amazonensis*, for instance, secretes NDK during infection, preventing ATP-mediated cytolysis of macrophages [3]. Therefore, this multifunctional enzyme also works on preserving the integrity of host cells to benefit the parasites [3].

Despite the high similarity in amino acid sequence, NDKs can assume different quaternary arrangements. Most eukaryotic NDKs form hexamers while some bacterial enzymes form tetramers [16-18]. The main difference between tetrameric and hexameric NDKs relies on their C-terminal region. In tetrameric NDKs, the C-terminal extension interacts with the neighboring subunit of the same dimer, whereas in hexameric NDKs this region interacts with the adjacent dimer, contributing for hexamer stability [19].

In hexameric NDKs, the quaternary structure is important for enzymatic activity [19]. However little is known about how environment conditions such as ionic strength and pH affect their oligomeric stability. It has been demonstrated that salt concentration modulate hexameric assembly and activity of a halophilic NDK [20], but the influence of pH in hexameric NDKs stability remains elusive. Here we report the crystal structure and spectroscopic characterization of *L. braziliensis* NDK (*Lb*NDK) under distinct pH conditions similar to that faced by the parasite in the macrophages [21]. Our data shed light on conformational changes associated with acidic conditions, which decrease hexamer stability and reveal that the C-terminal extension of *Lb*NDK is partially unstructured, an unusual feature among eukaryotic NDKs.

## Results and Discussion

### Overall structure and interfaces description

*Lb*NDK crystals belonged to the space group *P*2_1_3 with a dimer in the asymmetric unit. Refinement converged to a crystallographic residual of 17% (*R*_free_ = 22%) and the final model resulted in good stereochemistry according to the Ramachandran plot and r.m.s.d. values of bond lengths and angles (Table 1).

**Table 1:** **Data processing and structure refinement statistics**

**Data collection**	
Space group	P2_1_3
Cell dimensions	
*a*, *b*, *c* (Å)	110.28
Resolution (Å)^#^	50.00-2.70 (2.80-2.70)
*R*_merge_ (%)	8.8 (54.8)
*<I* / σ*I>*	23.92 (4.13)
Completeness (%)	100 (100)
Multiplicity	8.1 (8.3)
**Refinement**	
Resolution (Å)	49.32-2.70
Number of reflections	12544
*R*_work_/*R*_free_	0.17/0.22
Number of atoms	
Protein	2171
Ligand/ion	10
Water	55
*B*-factor (Å^2^)	
Protein	53.7
Ligand/ion	57.5
Water	43.1
R.m.s. deviation	
Bond length (Å)	0.008
Bond angle (°)	1.117
Ramachandran	
Favored (%)	98.9
Allowed (%)	1.1
Disallowed (%)	0

^#^Values in parentheses are for the highest resolution shell.

**Figure 1:**
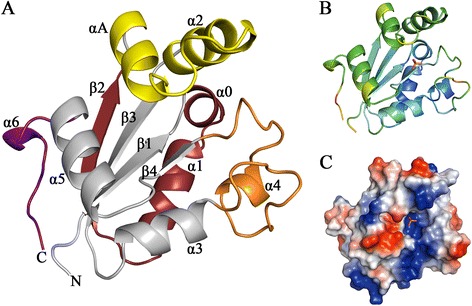
***Lb*** **NDK structure. (A)** Cartoon representation of *Lb*NDK protomer, showing secondary structure elements labeled according to the nomenclature proposed by Morera *et al.* [17]. Colors are used to highlight important regions: Kpn loop (orange), Head (yellow), C-terminal extension (purple) and dimer/trimer interfaces (red). **(B)** Cartoon representation of *Lb*NDK subunit, colored according to the B-factor values, from blue (lowest) to red (highest). **(C)** Electrostatic surface representation colored by charge from red (negative) to blue (positive) generated using the PyMOL Charge-smoothed potential approach. Inside the highly positive active site cleft, a phosphate ion (stick representation) is bounded to Lys^11^, Asn^114^ and the conserved residue His^117^.

The *Lb*NDK monomer consists of six α-helices partially involving a core of four β-strands arranged in a β2β3β1β4 antiparallel topology order, as observed in canonical NDK structures (Figure 1A) [17,22]. Interestingly, this fold is recurrent in different nucleotide-binding proteins [22-24]. B-factor analysis indicates that the N- and C-terminal extremities as well as regions not involved in dimer/trimer interfaces are the most flexible (Figure 1A and B). The highest flexibility comes from the C-terminal extension, whose last 9 residues in chain A and 12 residues in chain B were completely disordered and therefore were not modelled.

*Lb*NDK conserves the proline residue (Pro^95^) from the Kpn-loop (*Killer of prune*) involved in NDK stability [25,26]. Together with a region named Head (44–68) [17], the Kpn-loop (92–116) forms a cleft that harbors the highly positively-charged active site (Figure 1A and C), required for recognition and binding of negatively-charged substrates. As demonstrated for other NDKs, *Lb*NDK also conserves the key residue His^117^, which is essential for phosphate transfer [6], Tyr^51^, important for catalytic mechanism and Phe^59^ that stacks with the base of nucleotide substrates [27]. Other 13 residues already described as important for catalysis in homolog proteins are present as well [28].

**Figure 2:**
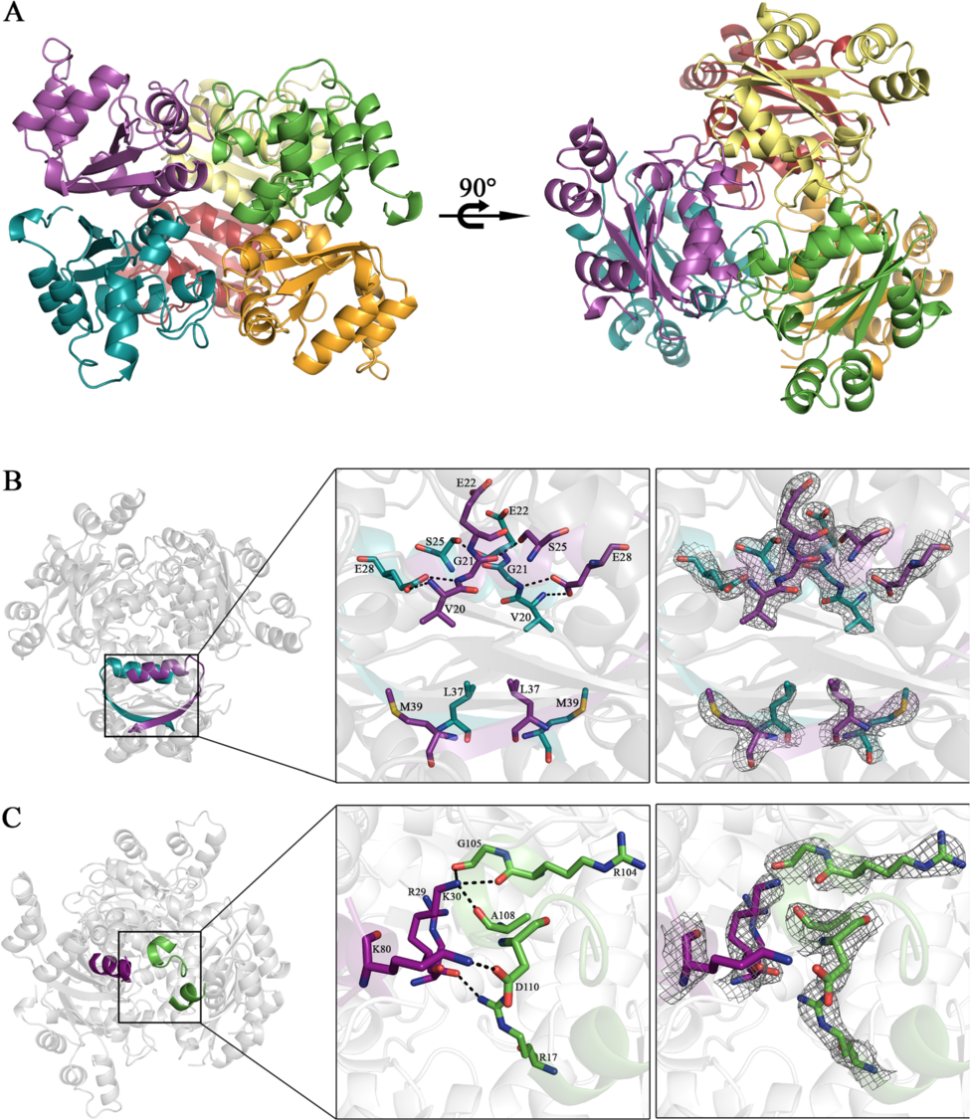
***Lb*** **NDK hexamer interfaces. (A)** Side and top views of the hexamer, with each subunit in a different color (red, orange and cyan in the bottom trimer; purple, yellow and green in the top trimer). **(B)** Dimeric interface regions from two different subunits (purple and cyan) with an inset corresponding to the zoom view that shows residues (sticks) involved in dimer stabilization via hydrogen bonds (dotted black lines) and the electronic density map for these residues. **(C)** Trimeric interface region between the purple and green subunits highlighting residues (sticks) that make hydrogen bonds (dotted black lines) and the electronic density map for these residues.

Analysis of symmetry-related chains in the crystalline unit cell revealed a hexameric arrangement similar to that observed for *Lm*NDK [28]. The hexamer can be seen as a trimer of dimers (Figure 2A). The dimer interface (712.4 Å^2^) is more extensive than the trimer interface (467.9 Å^2^) and comprises residues mainly from α1 and β2 elements (Figure 2B). The O^ε1^ and O^ε2^ atoms of Glu^28^ act as key hydrogen bond acceptors, contributing for dimer stabilization via hydrogen bonds with main-chain nitrogen atoms of Val^20^ and Gly^21^ from the interfacing subunit (Figure 2B). Additional hydrophobic interactions involving the residues Ala^139^ and Trp^141^ located at the C-terminal extension also stabilize the dimer. These residues interact with Val^15^, Met^39^ and Pro^71^ from the adjacent subunit, restricting solvent accessibility to the surface area buried in this interface.

In the trimer interface, residues located at α1 and β3-α3 loop make hydrogen bonds with residues located in the α0 and Kpn-loop from the other subunit (Figure 2C). The Lys^30^N^ζ^ atom is the main hydrogen bond donor to carbonyl oxygens from Arg^104^, Gly^105^ and Ala^108^ residues, contributing for trimer stabilization along with the Lys^80^-Asp^110^ ionic interaction and the hydrogen bond between Arg^17^ side chain and Arg^29^ backbone (Figure 2C).

### *Lb*NDK displays an unstructured C-terminus

**Figure 3:**
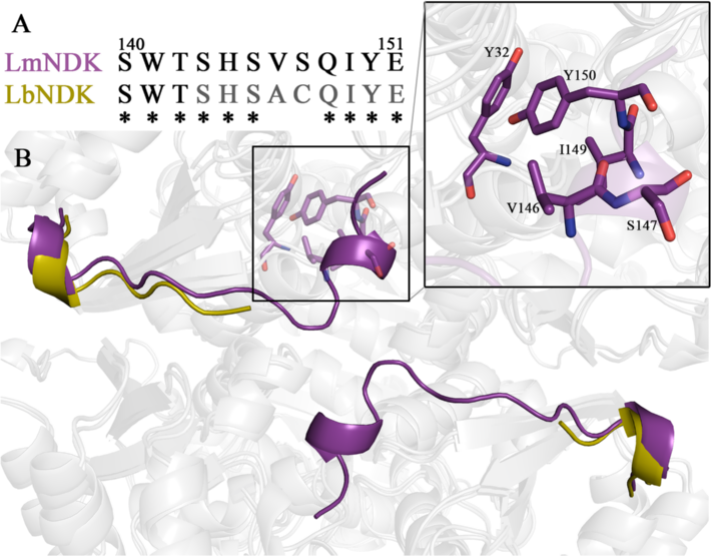
**Structure and sequence comparisons between** ***Lb*** **NDK and** ***Lm*** **NDK. (A)** CLUSTALW alignment of the C-terminal sequence from *Lm*NDK and *Lb*NDK. The region not modelled in the *Lb*NDK crystal structure (gray) contains only two amino acids residues (Ala^146^ and Cys^147^) that are not conserved in *Lm*NDK (Val^146^ and Ser^147^). **(B)** Cartoon representation of *Lb*NDK structure superimposed in the *Lm*NDK structure highlighting the C-terminal extension partially unfolded in *Lb*NDK (modelled up to residue 142 and colored in yellow) and fully folded in *Lm*NDK (purple). In sticks (purple) are residues that form a hydrophobic cluster that stabilize the C-terminal extension of *Lm*NDK and Ser^147^ that is substituted by a cysteine in *Lb*NDK.

*Lb*NDK and homolog *Lm*NDK (PDB code: 3NGT) monomers superimpose with an r.m.s.d. of 0.649 Å for 141 Cα atoms aligned and a sequence identity of 91.5%. The main structural difference is in the last 9 residues of the C-terminal extension. In *Lm*NDK, these residues are ordered and some of them contribute for hexamer stabilization, such as His^144^ at the dimer interface and Ile^149^, Tyr^150^ and Glu^151^ at the trimer interface. Intramolecular interactions of Val^146^ with Tyr^32^, Ile^149^ and Tyr^150^ contribute to maintain the *Lm*NDK C-terminus structured (Figure 3). Their side chains form a hydrophobic cluster that keeps Tyr^150^ tied to its own subunit surface. By comparing the C-terminal sequence of *Lb*NDK and *Lm*NDK, we observed only two residue substitutions. Replacement of *Lm*NDK Val^146^ and Ser^147^ residues by an alanine and a cysteine, respectively, might account for the destabilization of *Lb*NDK C-terminus (Figure 3A).

Since Ile^149^, Tyr^150^ and Glu^151^ compose the trimer interface in *Lm*NDK, the lack of these interactions in *Lb*NDK may influence the hexamer stability. Indeed, PDBePISA analysis of *Lb*NDK and *Lm*NDK interfaces indicate that the *Lm*NDK trimer interface is energetically more stable (ΔG = −2.6 kcal/mol) than that of *Lb*NDK (ΔG = −0.4 kcal/mol). The trimer interface area of *Lm*NDK, whose C-terminus is structured, is 727.9 Å^2^ whereas in *Lb*NDK, whose C-terminus is disordered, it is reduced to 467.9 Å^2^. Thus, absence of a well-structured C-terminal extension seems also to affect the hexameric stability. It is known that the deletion of 1–5 residues at the C-terminus of the NDK from *Dictyostelium* severely decrease its thermal tolerance [29], reinforcing the importance of this region for the structure.

The presence of an unstructured C-terminus is an atypical feature among eukaryotic NDKs. From the deposited three-dimensional structures in the PDB, only the human NDK 4 (*nmr23*-H4) [30] shares this feature with *Lb*NDK, according to the structure similarity searches using the PDBeFold service [31]. Besides the probable influence in hexamer stability, the lack of the C-terminus interactions at the trimer interface exposes a surface path formed by Pro^12^, Asp^13^, Gln^16^, Arg^17^ and the region from Val^109^ to Arg^113^ that might be explored for the rational design of compounds to inhibit hexamer formation, considered essential for enzymatic activity.

### *Lb*NDK is a hexamer at a broad pH range

In order to investigate whether the crystallographic hexamer is the oligomeric state of *Lb*NDK in solution at different pH conditions, dynamic light scattering (DLS) and small angle X-ray scattering (SAXS) experiments were performed. DLS assays showed monodisperse populations (Pd < 13%) with average hydrodynamic radius (*R*_*h*_) varying between 44 and 46 Å in the pH range from 4.0 to 9.0 (Table 2). The molecular masses estimated in these conditions are similar to the theoretical mass of the hexamer (112 kDa), indicating that *Lb*NDK maintains the hexameric arrangement either upon medium acidification or alkalization (Table 2).

**Table 2:** **Structural and hydrodynamic parameters calculated from DLS and SAXS experiments under different pH conditions**

	**pH**	**4.0**	**5.0**	**6.0**	**7.0**	**8.0**	**9.0**
**DLS**	*R*_h_ (Å)	46	46	44	44	44	45
	MW (kDa)	120	117	106	108	109	106
	% Pd	12.6	10.1	12	11.5	11.4	10.8
**SAXS**	*R*_g_ (Å)	34	33	32	31	-	-
	*D*_max_ (Å)	86	85	83	84	-	-
	MW (kDa)	110	110	109	109	-	-

*R*_h_ is the hydrodynamic radius, MW is the calculated molecular weight, % Pd corresponds to the sample polydispersity, *R*_g_ is the radius of gyration estimated by the Guinier approximation and *D*_max_ is the maximum molecule diameter estimated from the pair-distance distribution function *P*(*r*).

**Figure 4:**
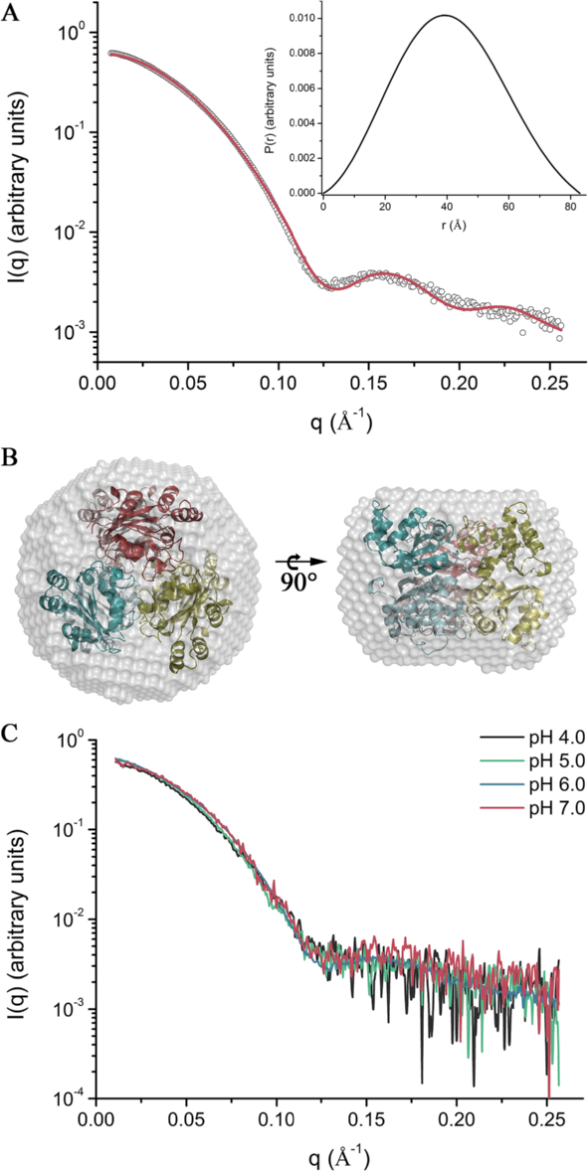
**Analysis of** ***Lb*** **NDK by SAXS. (A)** Experimental scattering curve (open dots) compared to the theoretical curve calculated for the crystallographic hexamer (red solid line). The inset shows the pair-distance distribution curve *P*(*r*) obtained from the experimental data. **(B)** *Lb*NDK crystallographic hexamer fitted into the low resolution SAXS envelope shown on top and side orientations. Hexamer can be viewed as a combination of top (dark colors) and bottom (light colors) trimers or as three dimers (blue, yellow and red shades). **(C)** Experimental scattering curves in pH 4.0 (black line), 5.0 (green line), 6.0 (blue line) and 7.0 (red line), showing that no significant changes occur on the *Lb*NDK quaternary structure at this pH range.

SAXS experiments performed at pH 4.0, 5.0, 6.0 and 7.0 showed similar results, regardless the pH condition, corroborating DLS data (Table 2). From the scattering and pair-distance distribution curves at pH 6.0, the maximum molecular dimension (*D*_max_) was determined as 83 Å with a radius of gyration of 32 Å and a calculated molecular mass of 109 kDa, which is accordance with the theoretical mass of the hexamer (Table 2 and Figure 4A). Moreover, the hexameric crystal structure showed a good agreement with the molecular envelope generated from SAXS data by *ab initio* calculations, supporting that the crystallographic hexamer corresponds to the oligomeric state assumed by *Lb*NDK in solution (Figure 4B). The pH decrease from 7.0 to 4.0 did not altered significantly the scattering curves of *Lb*NDK (Figure 4C), indicating that the enzyme remains hexameric in pH conditions akin to that faced by the enzyme secreted by *Leishmania* spp. in the parasitophorous vacuoles (pH 4.7 to 5.3) [32].

### Conformational stability under distinct pH conditions

**Figure 5:**
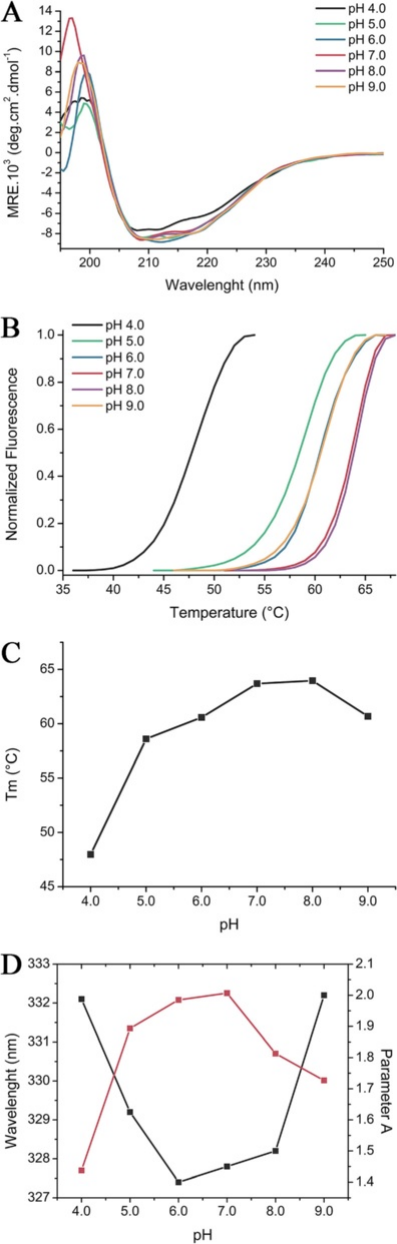
**CD, DSF and ITFE measurements under different pH conditions.** Data were collected at pH 4.0 (black line), 5.0 (green line), 6.0 (blue line), 7.0 (red line), 8.0 (purple line) and 9.0 (orange line). **(A)** Far-UV CD spectra from 195 to 250 nm using 0.3 mg.mL^−1^ of protein sample. **(B)** Normalized thermal denaturation curves using SYPRO-Orange as the fluorescent probe. **(C)** Melting temperature (T_m_) calculated from the Boltzmann fit of denaturation curves as a function of pH. **(D)** Tryptophan maximum emission wavelength (black squares and black line) and Parameter A calculation analysis (red squares and red line) as a function of pH. *Lb*NDK (0.06 mg.mL^−1^) tryptophan emission was monitored from 300 to 450 nm using an excitation wavelength of 295 nm.

To gain insights into the effect of pH in secondary structure and hexamer stability, circular dichroism (CD), differential scanning fluorimetry (DSF) and fluorescence experiments were performed. With decreasing pH, the CD spectra started to change, especially at pH 4.0, suggesting that acidification induces conformational changes in *Lb*NDK (Figure 5A). DSF assay pointed out that pH changes influence the *Lb*NDK thermal stability. *Lb*NDK presented considerably lower stability at pH 4.0 as showed by the negative T_m_ shifts of more than 10°C compared to the highest T_m_ (64°C), estimated at pH 7.0 (Figure 5B and C). Between pH 5.0 and 9.0, T_m_ shifts of about 5°C were observed, suggesting that *Lb*NDK presents similar thermal stability at this pH range (Figure 5B and C).

Moreover, the single transition of the thermal denaturation curves (Figure 5B) suggests that the denaturation does not occur by discretized steps involving hexamer dissociation followed by monomer unfolding. This result also agrees with SAXS and DLS assays, which indicates that the quaternary structure is maintained even at acidic or alkaline conditions (Table 2). The single transition was also described for tetrameric and hexameric NDKs from different organisms, indicating a similar thermodynamic denaturation process among these enzymes [18].

*Lb*NDK has three tryptophan residues, at positions 77, 132 and 141, and the intrinsic fluorescence tryptophan emission (IFTE) from those residues provided information about variations in their microenvironment, consequent from conformational changes induced by pH variations. At neutral pH, ITFE of *Lb*NDK presented a λ_max_ near to 328 nm (Figure 5D, black line), which shifted to near 332 nm at pH 4.0 and 9.0, suggesting that either acidification or alkalization induce conformational changes that expose one or more tryptophan residues to a more polar environment. Analysis of the parameter A indicates that *Lb*NDK maximal stability is reached between pH 6.0 and pH 7.0 (Figure 5D, red line). These data are consistent with a permanent occlusion of tryptophans in protein structure at this pH range. Parameter A decreased both with increasing and decreasing pH, further supporting that conformational changes occur upon medium acidification or alkalization (Figure 5D, red line).

**Figure 6:**
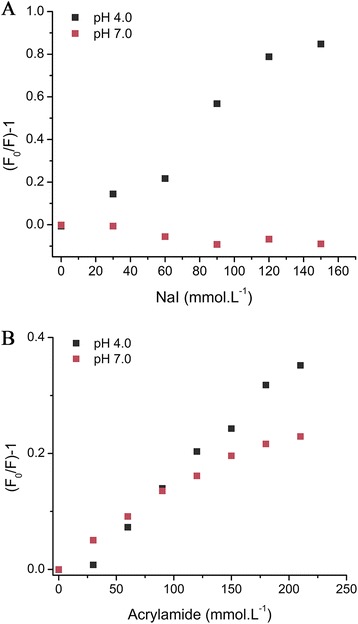
**Analysis of** ***Lb*** **NDK tryptophan fluorescence quenching.** Modified Stern-Volmer plots for quenchers **(A)** iodide and **(B)** acrylamide, both at pH 4.0 (black squares) and 7.0 (red squares). Protein at 0.06 mg.mL^−1^ was excited at 295 nm and data collected from 300 to 450 nm.

Fluorescence quenching studies were also carried out to understand tryptophans microenvironments under neutral to acidic conditions. The Stern-Volmer plots for quenching of *Lb*NDK tryptophans by iodide (surface quencher) and acrylamide (neutral internal quencher) are shown in Figure 6.

When using iodide at pH 7.0, no quenching is observed, indicating that Trp^77^, Trp^132^ and Trp^141^ are buried in the hydrophobic core of *Lb*NDK structure under this condition (Figure 6A). The switching to acidic pH results in increasing values of [(F_0_/F) – 1] in function of NaI concentration, suggesting that structural changes increase iodide accessibility to one or more tryptophans. The non-linearity of the curve measured at pH 4.0 might be due to different levels of exposure of *Lb*NDK tryptophans to the solvent. Supporting this hypothesis, analysis of *Lb*NDK crystal structure shows that Trp^77^ is the most buried, with an accessible surface area (ASA) of 0.62 Å^2^ followed by Trp^132^ (ASA = 12.85 Å^2^) and Trp^141^. The latter was not modeled in the chain B of the crystallographic structure due to the high flexibility of the C-terminal extension, thus it was not considered for these calculations.

Results are slightly different using the quencher acrylamide (Figure 6B). As it can access less exposed residues, acrylamide quenching is already observed at pH 7.0. The quenching effects at pH 7.0 and pH 4.0 were very similar, suggesting that pH variations in this range are not sufficient to alter the acrylamide accessibility.

## Conclusions

Although acidification decreases the thermal stability of *Lb*NDK, inducing conformational changes that affects secondary structure and tryptophans microenvironment, it is not sufficient to dissociate the hexamer, supporting that *Lb*NDK remains hexameric in pH conditions akin to that faced by this enzyme when secreted by *Leishmania* amastigotes in the parasitophorous vacuoles (pH 4.7 to 5.3) [32]. Differently from many eukaryotic NDKs, *Lb*NDK displays an unstructured C-terminus that exposes a surface path in the trimer interface. As the quaternary structure of NDKs is essential for full enzymatic activity [33], the rational design and development of compounds targeting this exposed region may be a valuable strategy to discover new anti-leishmanial drugs.

## Methods

### Cloning, expression and purification

*L. braziliensis* NDK (*Lb*NDK) open read frame (Lmjf_32_2950) was amplified by polymerase chain reaction (PCR) using genomic DNA as template and cloned into the expression vector pET28a (Novagen) using the *Nde*I and *BamH*I restriction sites. The recombinant *Lb*NDK fused to an N-terminal His-tag was produced in the *E. coli* BL21 (DE3) pLysS strain grown at 37°C in 750 mL HDM medium containing 10 mmol.L^−1^ MgSO_4_ and 50 μg.mL^−1^ kanamycin. Overexpression was induced with 0.6 mmol.L^−1^ isopropyl β-D-1-thiogalactopyranoside (IPTG, Promega) when the culture reached an OD_600nm_ of 0.6. After 5 hours, the cells were harvested by centrifugation at 5000 × g for 10 min at 4°C. The cell pellet was resuspended in 40 ml lysis buffer (50 mmol.L^−1^ phosphate, 300 mmol.L^−1^ NaCl, 40 mmol.L^−1^ imidazole pH 8.0) containing 4 mmol.L^−1^ phenylmethylsulfonyl fluoride (PMSF, Sigma) and 1% *(v/v)* Triton X-100, sonicated for 10 × 30 s with 30 s interval between each pulse and centrifuged at 10000 × g for 30 min at 4°C. The supernatant was applied onto a HiTrap Chelating HP 5 mL column (GE Healthcare) pre-equilibrated with lysis buffer using an ÄKTA fast protein liquid-chromatography (FPLC) system (GE Healthcare). After washing the resin, the bound fractions were eluted using a linear gradient from 0 to 0.5 M imidazole in 20-column volume at a flow rate of 1 ml.min^−1^. The eluted protein was concentrated to 1.0 ml using an Amicon Ultra-4 10 K centrifugal device (Millipore) and loaded onto a HiLoad 16/60 Superdex 200 (GE Healthcare) size-exclusion column pre-equilibrated with 10 mmol.L^−1^ MES buffer pH 6.0 containing 50 mmol.L^−1^ NaCl, 10 mmol.L^−1^ MgCl_2_ and 2 mmol.L^−1^ dithiothreitol (DTT) at a flow rate of 0.5 ml.min^−1^. Fractions containing the protein were analyzed by SDS-PAGE 15% and stained with Coomassie brilliant blue R-250 (Sigma-Aldrich). Fractions with purity estimated to be superior to 99% were pooled and concentrated to 10 mg.ml^−1^. The protein concentration was estimated by UV absorbance at 280 nm using the theoretical extinction coefficient of 22,460 M^−1^ cm^−1^ calculated using ProtParam [34].

### Protein crystallization

Protein sample at 10 mg.mL^−1^ in 50 mmol.L^−1^ NaCl, 10 mmol.L^−1^ MgCl_2_, 2 mmol.L^−1^ DTT and 10 mmol.L^−1^ MES pH 6.0 buffer was used in crystallization experiments, performed by the sitting-drop vapor-diffusion method at 18°C using a Cartesian HoneyBee 963 system (Genomic Solutions). A total of 544 conditions from commercially available crystallization kits from Hamptom Research (SaltRx, Crystal Screen I and II), Emerald BioSystems (Precipitant Synergy and Wizard I and II) and Qiagen/Nextal (PACT and JCSG+) were tested. A drop of protein solution (0.5 μL) was mixed with the same volume of crystallization solution and equilibrated over a reservoir containing 80 μL of the latter solution. For crystal optimization, the initial condition was refined using a systematic grid in which sodium di-hydrogen phosphate concentration (from 0.8 mol.L^−1^ to 0.74 mol.L^−1^ in steps 20 mmol.L^−1^) was varied in function of di-potassium hydrogen phosphate concentration (from 1.2 mol.L^−1^ to 1.0 mol.L^−1^ in steps of 100 mmol.L^−1^) in 0.1 mol.L^−1^ sodium acetate buffer at pH 4.5. *In situ* proteolysis was also performed by adding trypsin at 1:100, 1:1,000 and 1:10,000 trypsin:*Lb*NDK ratio. A single crystal with approximate dimensions of 150 × 150 μm was obtained using a solution consisting of 0.78 mol.L^−1^ sodium di-hydrogen phosphate, 1 mol.L^−1^ di-potassium hydrogen phosphate, 0.1 mol.L^−1^ sodium acetate (pH 4.5) and 1:10,000 trypsin:*Lb*NDK ratio. The final pH of the crystallization condition was around 7.0 due to the presence of the high concentration of phosphate.

### X-ray data collection, processing and structure determination

Diffraction data were collected at the W01B-MX2 beamline from the Brazilian Synchrotron Light Laboratory (Campinas, Brazil). Crystals were soaked into a cryoprotectant solution (precipitant condition plus 30% *(v/v)* glycerol) for 30 s and then flash-cooled in a nitrogen gas stream at 100 K. The wavelength and the crystal-to-detector distance were set to 1.458 Å and 140 mm, respectively. X-ray diffraction data were recorded by a MarMosaic 225 CCD detector using an exposure time of 30 s and an oscillation angle of 1° per image. A total of 180 images were collected and the data were indexed, integrated and scaled using the HKL2000 package [35]. Molecular replacement was performed using the program MOLREP [36] and the atomic coordinates of NDK from *L. major* (PDB code 3NGS; [37]) as template. Refinement cycles were carried out using COOT [38] and PHENIX [39] programs. TLS-refinement was applied in the last cycles of refinement using TLS parameters from TLSMD server [40]. Model quality was evaluated using Molprobity [41]. Quaternary structure analyses were performed with the softwares PDBePISA [31] and Protein Interaction Calculator (PIC) [42]. Data collection and refinement statistics are shown in Table 1. The atomic coordinates have been deposited at the Protein Data Bank (PDB) under the accession code 4KPC.

### Dynamic Light Scattering (DLS)

DLS measurements were carried out using a DynaPro 810 (Protein Solutions, Wyatt Technology Corporation) system equipped with a temperature-controlled microsampler. An autopilot run with 100 measurements every 10 s was used at a constant temperature of 4°C and protein concentration of 1 mg.mL^−1^ in 20 mmol.L^−1^ of different pH buffers (acetate pH 4.0, citrate pH 5.0, MES pH 6.0, HEPES pH 7.0, Tris pH 8.0, and glycine/NaOH pH 9.0). The hydrodynamic parameters were determined using the Dynamics v.6.3.40 software. The hydrodynamic radius (*R*_h_) was extrapolated from the translational diffusion coefficient (Dt) using the Stokes–Einstein equation.

### Small angle X-ray scattering (SAXS)

*Lb*NDK at 2.0 mg.mL^−1^ was dialyzed overnight against 50 mmol.L^−1^ NaCl and 20 mmol.L^−1^ of different pH buffers (acetate pH 4.0, citrate pH 5.0, MES pH 6.0, and HEPES pH 7.0). SAXS data were collected at 12°C with exposure time of 100 s on the SAXS-1 beamline at the Brazilian Synchrotron Light Laboratory (Campinas, Brazil). The radiation wavelength was set to 1.55 Å and a PILATUS 300 K detector (DECTRIS) was used to record the scattering patterns. The sample-to-detector distance was set to 1564.817 mm to give a range of the scattering vector q from 0.008 to 0.25 Å^−1^, where q is the magnitude of the q-vector, defined by q = 4π sinθ/λ and 2θ is the scattering angle. SAXS patterns were integrated using the Fit2D software [43]. The experimental radius of gyration (*R*_g_) was computed using the program AUTORG [44]. Data fitting and evaluation of the pair-distance distribution function *P*(*r*) was performed using the program GNOM [45]. *Ab initio* low resolution models were calculated from the scattering data using the software DAMMIN [46] and averaged from several runs using the software DAMAVER [47]. The theoretical scattering curve and *R*_g_ were calculated from atomic coordinates using the software CRYSOL [48].

### Circular dichroism (CD)

The circular dichroism spectra of *Lb*NDK (0.3 mg.mL^−1^) were recorded between 190–250 nm in a spectropolarimeter JASCO810 (JASCO Inc.) using a 0.1 cm quartz cuvette. Each CD spectrum accumulates five scans at 50 nm.min^−1^ with a 1 nm width slit and 1 s response. The measurements were carried out in 20 mmol.L^−1^ of several buffers (phosphate/citrate pH 4.0, 5.0, 6.0 and 7.0; glycine/NaOH 8.0 and 9.0). All spectra were corrected for the buffer contributions and converted to MRE (mean residue ellipticity) in deg.cm^2^.dmol^−1^, defined as:

1 $$ MRE=\frac{M\theta }{10.d.c.r} $$

where M is the molecular weight of the protein, θ is the ellipticity in millidegrees, d is the optical path in cm, c is the concentration of the protein sample in mg.mL^−1^ and r is the estimated number of residues in the analyzed protein.

### Differential Scanning Fluorimetry (DSF)

*Lb*NDK were incubated overnight in 20 mmol.L^−1^ of different buffers (acetate pH 4.0, citrate pH 5.0, MES pH 6.0, HEPES pH 7.0, Tris pH 8.0, and glycine/NaOH pH 9.0) and assayed at a final concentration of 2.0 μmol.L^−1^ in 25 μL total volume. SYPRO-Orange (Invitrogen Molecular Probes) was used as the fluorescence probe at a final 1:1000 dilution of a 5000× stock. Samples were heated at a rate of 1°C/min from 25 to 95°C and fluorescence emission was measured at 580 nm using a real time PCR machine 7300 (Applied Biosystems). The melting temperatures (T_m_) were calculated by fitting the melting curves with the Boltzmann equation.

### Intrinsic tryptophan fluorescence

The intrinsic tryptophan fluorescence emission was measured using a spectrofluorimeter HITACHI F-4500. The enzyme solution (0.06 mg.mL^−1^) was excited at 295 nm and the spectra obtained between 300–450 nm. Slits of 5 nm each were defined for the excitation and emission monochromators and the spectra collected at 240 nm/min. All spectra were corrected for the buffer contributions. Protein samples were incubated for 12 hours in different pH conditions (phosphate/citrate pH 4.0, 5.0, 6.0 and 7.0; and glycine/NaOH 8.0 and 9.0) before data acquisition, using the same buffers described for CD measurements. Parameter A, the ratio between the intrinsic fluorescence intensities at 320 nm and 365 nm, was also calculated, since it is a sensitive indicator of structural changes of proteins during induced denaturing assays [49].

### Intrinsic tryptophan fluorescence quenching

Fluorescence quenching measurements were carried on HITACHI-4500 spectrofluorimeter at 25°C in a 1.0 cm quartz cuvette. Protein solution was excited at 295 nm and emission spectra were obtained between 300 – 450 nm. Slits of 5 nm each were defined for the excitation and emission monochromators and the spectra collected at 240 nm.min^−1^. All spectra were corrected for buffer contributions. Protein were incubated for 12 hours at concentration 0.06 mg.mL^−1^ in buffer containing 20 mmol.L^−1^ phosphate/citrate pH 4.0 or 7.0, each with 150 mmol.L^−1^ NaCl. Two quenchers were utilized: acrylamide at concentration range from 0 to 210 mmol.L^−1^, varying 30 mmol.L^−1^ and NaI at concentration range from 0 to 150 mmol.L^−1^, varying 30 mmol.L^−1^. Fluorescence quenching was evaluated by plotting (F_0_/F) - 1 in function of quencher concentration. F_0_ and F are the integrated fluorescence emission intensities in the absence and presence of increasing quencher concentration, respectively.

### Availability of supporting data

The data set supporting the results of this article are available in the Protein Data Bank repository, Accession Code 4KPC in http://www.rcsb.org/pdb/explore/explore.do?structureId=4KPC.

## Abbreviations

NDK: Nucleoside diphosphate kinase
LmNDK: NDK from *Leishmania major*
LbNDK: NDK from *Leishmania braziliensis*
nmr23-H4: NDK 4 from *Homo sapiens*
Kpn: Killer of prune
NTP: Nucleoside triphosphate
ATP: Adenosine triphosphate
PMSF: Phenylmethylsulfonyl fluoride
DTT: Dithiothreitol
IPTG: Isopropyl β-D-1-thiogalactopyranoside
HDM: High density medium
DLS: Dynamic light scattering
SAXS: Small angle x-ray scattering
DSF: Differential scanning fluorimetry
CD: Circular dichroism
IFTE: Intrinsic fluorescence tryptophan emission
r.m.s.d: Root mean square deviation
Pd/% Pd: Sample polidispersivity
*R*_*h*_: Hydrodynamic radius
Dt: Translational diffusion coefficient
*D*_max_: Maximum molecular dimension
T_m_: Melting temperature
λ_max_: Maximum wavelenght
*R*_g_: Radius of gyration
*P*(*r*): Pair distance distribution function
2θ: Scattering angle
MRE: Mean residue elliptcity
M/MW: Molecular weight
θ: Ellipticity
d: Optical path
c: Protein concentration
r: Estimated number of residues
F: Integrated fluorescence emission intensities in the presence of the quencher
F_0_: Integrated fluorescence emission intensities in the absence of the quencher
q: Magnitude of the scattering vector
ΔG: Gibbs’ free energy variation
ASA: Accessible surface area
PDB: Protein data bank
PIC: Protein interface calculator
UV: Ultraviolet
